# Validating interRAI Chinese self-reported carer needs (SCaN) assessment and predicting caregiving distress among informal Chinese caregivers of older adults

**DOI:** 10.1186/s12877-024-05014-0

**Published:** 2024-05-08

**Authors:** Shicheng Xu, Vivian W. Q. Lou, Iris Chi, Wai Chong Ng, Jing Zhou, Lung-Kuan Huang, Carol Hok Ka Ma, Moana Jagasia

**Affiliations:** 1 Sau Po Centre on Ageing, The University of Hong Kong, Hong Kong, China; 2 Department of Social Work & Social Administration, The University of Hong Kong, Hong Kong, China; 3 Suzanne Dworak-Peck School of Social Work, University of Southern California, Los Angeles, USA; 4 NWC Longevity Practice, Clementi, Singapore; 5 Department of Social Sciences, Faculty of Social Sciences and Business Studies, University of Eastern Finland, Joensuu, Finland; 6 Department of Social Sciences, Faculty of Social Sciences and Business Studies, Shanghai Lixin University of Accounting and Finance, Shanghai, China; 7 St. Camillus Long-term Care Center, Yi-lan County, Taiwan; 8 Fo Guang University, Yi-lan County, Taiwan; 9 Singapore University of Social Science, Clementi, Singapore

**Keywords:** Psychometrics, Informal caregiver, Caregiving distress, Chinese caregivers, Assessment

## Abstract

**Background:**

This study aims to (1) determine the reliability and validity of the interRAI Chinese Self-reported Carer Needs (SCaN) assessment among informal Chinese caregivers of older adults, (2) identify predictors of caregiving distress in Asian regions with long-standing Confucian values of filial piety and family responsibility.

**Methods:**

This cross-sectional study recruited 531 informal Chinese caregivers of older adults in Hong Kong, Shanghai, Taiwan, and Singapore. The scale reliability was examined using Cronbach’s alphas (α) and McDonald’s omega coefficient (ω). The concurrent validity and discriminant validity were assessed using Spearman rank correlations (*rho*). To examine the predictors of caregiving distress among informal caregivers of older adults, we employed hierarchical linear regression analyses informed by the Model of Carer Stress and Burden and categorized the predictors into six domains.

**Results:**

Results revealed good internal consistency reliability (α = 0.83–0.96) and concurrent validity (*rho* = 0.45–0.74) of the interRAI Chinese SCaN assessment. Hierarchical linear regression analysis revealed that entering the background factors, primary stressors, secondary stressors, appraisal, and exacerbating factors all significantly enhanced the model’s predictability, indicating that the source of caregiving distress is multidimensional. In the full model, caregivers with longer informal care time, lack of support from family and friends, have unmet needs, experience role overload, have sleep problems, and low IADL functioning are at a higher risk of caregiving distress.

**Conclusions:**

The interRAI Chinese SCaN Assessment was found to be a reliable and valid tool among the Chinese informal caregivers of older adults. It would be useful for determining family caregivers’ strengths, needs, and challenges, and tailoring interventions that address the potentially modifiable factors associated with caregiving distress and maximize support. Healthcare providers working in home and community settings should be aware of the early identification of caregiving distress and routine assessment of their needs and empower them to continue taking care of their needs and providing adequate care to the care recipient.

**Supplementary Information:**

The online version contains supplementary material available at 10.1186/s12877-024-05014-0.

The world population is ageing rapidly, resulting in rising long-term care demands and healthcare costs, which in turn lead to a shift from formal to informal care [1]. Supporting informal caregivers thus becomes a crucial public health issue worldwide [2]. An informal caregiver is a relative, spouse, partner, or friend who provides care and support to someone at home without pay [3]. They are viewed as a valuable extension of the healthcare system and the first line of support for older people with medical, behavioral, disability, or other conditions [4]. However, despite the important role of informal caregivers in long-term care provision, their needs are poorly understood and largely remain under-recognized by service providers [5]. In the past decade, the complexity of caregiving has increased, requiring informal caregivers to acquire a sophisticated understanding of the care recipients’ conditions and new skills, but oftentimes, they are not adequately trained or prepared, and lack of choice in taking on the role [2, 6]. Meanwhile, informal caregivers of older adults are at an increased risk of experiencing distress, resulting in physical and mental health problems and discontinuation of caregiving, which may negatively impact patient outcomes, leading to hospitalization and nursing home placement [7–10].

*Caregiving distress* is the psychological distress associated with caring for a chronically ill individual, which is distinct from the objective and tangible costs indicated by *caregiving burden* [11]. Measuring the subjective experience of caregiving is arguably more predictive of caregiver health and well-being [12]. However, to date, the caregiving distress process identified in the literature is mostly specific to caregivers of people with a particular condition, such as cancer and dementia, and not generalizable across conditions and caregiver contexts [8, 13]. Identifying and addressing potentially modifiable factors contributing to caregivers’ distress are much needed to ensure their health, well-being, and ability to remain in the caregiving role [14].

Asia is ageing faster than any other continent [15]. By 2030, Asia will be the region with the largest elderly population in the world, exceeding 4.9 billion, and the informal caregiving demand will continue to increase [16]. Compared to developed countries, most developing countries in Asia lack structured health schemes for older adults, resulting in a higher prevalence of family caregiving for older adults [17]. However, due to the rapid socio-economic and demographic changes, Asia has witnessed an upsurge of nuclear families, and the reliance on the care provided by families has become untenable [18]. Caregiving in Asia is further complicated by the long-standing Confucian values of filial piety and family responsibility [19, 20]. Originating in China, Confucianism is still predominant in many Chinese-majority societies, including mainland China, Hong Kong, Taiwan, and Singapore [12]. Filial piety prescribes that adult children provide care, respect, and financial support to their older parents [21]. However, such sociocultural values of family caregiving can be burdensome [22]. The cultural sense of family obligation may prevent caregivers from seeking help outside of the household despite support is much needed [23]. Consequently, their needs are neglected, and health is compromised, and informal caregivers of Chinese ethnicities are susceptible to increased risks of caregiving distress [24]. Therefore, it is imperative to validate internationally recognized assessment tools that can measure the needs and distress perceptions of Chinese caregivers, facilitating cross-cultural comparisons, and enabling the development of interventions and support services that are sensitive to cultural differences [25].

In an international effort to support assessment, care planning, outcome evaluation, and resource allocation, interRAI is a collaborative network of researchers and practitioners in over 35 countries committed to improving care for persons who are disabled or medically complex (www.interRAI.org). A family of standardized assessment tools have been developed and rigorously tested for vulnerable older adults in long-term care homes, home care, acute care hospitals, rehabilitation, and palliative care since 1992. These tools have also been used to investigate predictors of caregiver distress using patients’ assessments, such as interRAI Home Care and interRAI Palliative Care. However, with only a few items dealing with informal caregivers, most predictors are derived from the patient’s conditions [14, 26]. Therefore, introducing a standardized, self-reported caregiver supplement to the current interRAI suite would help understand their source of distress, needs and challenges, and navigate support services [26]. Routine screening and assessment of psychological and physical health needs, as well as preventive measures oriented towards informal caregivers across their caregiving journey, should be core elements of optimal family-centered and community-based care [2, 3, 6, 10, 17, 27–29].

In recent years, interRAI developed a comprehensive assessment tool, interRAI Self-Reported Carer Needs (SCaN) assessment, to systematically gather information from the caregivers’ perspective about 1) their unmet financial, physical, emotional, or social needs; (b) their emotional and physical functioning; (c) their ability to realistically provide care while still be involved in other activities; and (d) services that best match their unique needs, challenges, and expectations. As part of interRAI’s multinational research initiative, four interRAI Asian members, Shanghai (in mainland China), Hong Kong, Taiwan, and Singapore collaborated to explore the applicability of the interRAI SCaN assessment among Chinese caregivers, as the four regions share a similar context of having a Chinese-majority population. To date, there is a lack of standardized assessment to examine the caregivers’ strengths, needs, and challenges among Chinese informal caregivers of older adults. The Model of Carer Stress and Burden (MCSB), derived from the classic Pearlin stress process model, is one of the main models used to explain negative caregiving outcomes. It assumes the risk and protective factors identified in caregiving situations can be categorized into six distinct domains (background factors of stress, primary stressors, secondary stressors, appraisal, and exacerbating factors), each with a unique contribution to caregiving distress [30, 31]. As the predictors of caregiving distress across conditions and caregiver contexts have not been systematically reported in the Asian context, this study will apply MCSB to identify and stratify the level of caregiving distress predictors in the interRAI Chinese SCaN assessment. In summary, the present study aims to (1) translate and determine the reliability and validity of the Chinese SCaN assessment among informal caregivers of older adults in communities with a Chinese-majority population; (2) identify the predictors of caregiver’s distress among informal Chinese caregivers of older adults.

## Methods

### Settings

This study involves informal caregivers of older adults in four Asian sites: Hong Kong, Shanghai, Taiwan and Singapore (Singapore, 75.9% of the population is Chinese people, [32]). Despite the diverse sociopolitical structures, the four study sites all comprise a majority of the Chinese population and share Confucian cultural values [33]. Families in Hong Kong, Shanghai, Taiwan, and Singapore are all considered central in caring for frail older people [22, 34–36]. The four sites also face similar challenges in family care provision, such as declining fertility rates, increased life expectancy and old-age dependency ratio, family downsizing and reduced intergenerational cohabitation, and concomitantly increased informal caregiving demands [34]. Thus, exploring their needs, challenges, and strengths can provide a comprehensive understanding of the caregiving experience within the broader Chinese cultural context.

### Sampling and participants

Power analysis indicates that the optimal sample size in each group is 45 (group k = 4, effect size f = 0.25, alpha = 0.05). To account for missing data, we will oversample by 10%, thus each region will recruit 50 to 100 caregivers, resulting in a final sample size of 200 to 400 survey respondents. The inclusion criteria of informal caregivers are: (1) aged 21 or above; (2) understanding written Chinese; (3) caring for older adults aged 60 years or above (can be family or non-relative); (4) primary caregivers, unpaid for their service and providing most of the care. The exclusion criteria are: (1) self-reported cognitive or mental health issues; (2) the care recipient lives in institutional settings, where care is provided by professionals and paid staff. All four study sites used convenience sampling with the same inclusion and exclusion criteria to ensure the consistency.

### Data collection procedures

This study uses self-reported survey data collected from January to March 2023. Currently, there is only an English version of the interRAI SCaN assessment. Considering the needs of Chinese-speaking caregivers, the original questionnaire was translated into Chinese by two Hong Kong-based bilingual research assistants with social work backgrounds and then using the back-translation procedures proposed by Brislin and Freimanis [37]. The Hong Kong version was further slightly revised and modified by Shanghai, Taiwan, and Singapore investigators as the health care system and the use of the Chinese language were different in different Chinese societies. For instance, despite Chinese being the common language across four regions, Cantonese is the main dialect in Hong Kong in the form of traditional Chinese, whereas most Taiwanese speak Mandarin but use traditional Chinese as their written language. In comparison, Shanghai and Singaporean Chinese also speak Mandarin but use simplified Chinese as the written language. Also, a few vocabularies, grammar, and syntax were changed, resulting in three versions of interRAI Chinese SCaN assessments (Hong Kong Cantonese version, Taiwan traditional Chinese version, Shanghai simplified Chinese version). The translated assessments were then piloted among five caregivers in each site and minor adjustments were made to improve understandability and clarity. All principal investigators administered the translation and examined the face validity and content validity of the translated versions.

InterRAI SCaN assessment is designed to be self-reported by caregivers as research has shown that most caregivers can complete the assessment independently (interRAI, 2022, p. 4). Therefore, the translated Chinese version of the interRAI SCaN assessment was completed via Qualtrics, an online surveying tool. The informed consent form was first obtained from all participants at the beginning of the survey. Participants were informed of the study objectives, and they had the right to withdraw from the study without any negative consequences. Only research team members will be granted access to the collected data.

The data collection approach was slightly different in the four study sites. Potential participants were either approached via social media or elderly care centers in the community, where they learned about the study from social workers. In Hong Kong, the research center distributed recruitment posters via social media to reach potentially eligible caregivers across the territory. Those interested and meeting the inclusion criteria could scan the QR code to complete the questionnaire. They were also encouraged to share the posters and links with others. Participants in Shanghai, Taiwan, and Singapore were approached in local elder care centers. Social workers were mainly responsible for introducing the study to caregivers and sending them the Qualtrics link. If the caregivers could not complete the questionnaire independently, the social workers would assist them or print out paper versions to facilitate the process.

The final sample size is 531 informal caregivers caring for older adults aged 60 years old and above. Sixty-four invalid cases were deleted as the age of care recipients is under 60. Thus, the valid response rate is 89.24%. The average time to complete an assessment was about 20 to 30 min. In our sample, 30.3% of caregivers were not digitally literate, interviewers thus guide them to use the digital platform, or they might be completely interviewed rather than self-report. This study was approved by the university’s Human Research Ethics Committee on July 4, 2022 (HREC Reference Number: EA220277).

## Measures

### Measurements for construct validity

Patient Health Questionnaire-4 (PHQ-4), Pearlin Role Overload Measure, and Lubben Social Network Scale (LSNS-6) will be used to measure construct validity. Caregivers are required to complete both the Chinese version of the interRAI SCaN assessment and the validation instruments.

#### Patient health questionnaire-4 (PHQ-4)

This is a valid ultra-brief questionnaire to detect both anxiety and depressive disorders [38]. PHQ-4 has also been validated in the Chinese context as a brief and valid measure of psychological distress [39]. It consists of a 2-item depression scale (PHQ-2) and a 2-item anxiety scale (GAD-2). Each item is rated on a 3-point Likert scale from 0 (not at all) to 3 (nearly every day).

#### Pearlin role overload measure

The 4-item Pearlin Role Overload Measure assesses the caregiver’s stress and constitutes not only the level of fatigue felt by caregivers but also the relentlessness and uncompromising nature of its sources [40]. The Chinese version was utilized by Cheng, Lam [41] among Hong Kong Chinese caregivers. Each item is rated on a 4-point Likert scale from 1 (not at all) to 4 (completely).

#### Lubben social network scale (LSNS-6)

This is a validated tool for assessing social networks and isolation among older Chinese by measuring the number and frequency of social contact with friends and family members and their perceived social support [42]. It consists of 6 items, and each question is scored from 0 (none) to 5 (nine).

### Independent predictors for caregiving distress

The interRAI Chinese SCaN Assessment offers a multidimensional perspective of the caregiving role. It is comprised of three sections: (1) demographic information; (2) carer health and wellbeing, including memory and cognition, social participation, function/endurance/stamina, mood, and health conditions (e.g., sleeping quality, pain, breath); (3) carer needs, including the supports needed and received from both caregivers and care recipients. It also identifies the challenges they encounter as an unpaid caregiver. Based on MCSB and the supporting evidence, twelve variables in the assessment were included in the multivariable regression models under five domains to predict caregiving distress (see Table S1 for background evidence that supports the inclusion of each factor). It hypothesized that each domain of stressors would significantly improve the predictability of the linear regression model for caregiver distress.

#### Background factors of stress

The caregiver’s year of birth, gender, and relationship to the care recipient were self-reported. The relationship factor includes the caregiver as the care recipient’s spouse/partner or child.

#### Primary stressors

Primary stressors include patient characteristics and care situations, and this study includes self-reported co-residence and informal care time as predictors. Co-residence is indicated by answering “We live together” to the time it takes to travel from the caregiver’s home to where the care recipient lives. Informal caregiving time is measured by the hours of care provided in the last three days, which was recoded into “less than 36 hours” and “36 hours or more”.

#### Secondary stressors

Secondary stressors arise from primary stressors, and this study includes financial difficulty and lack of social support as predictors. Financial difficulty is indicated by answering “yes” to “I have financial difficulties (e.g., have to make trade-offs using funds to cover food, shelter, clothing, or medications)”, and lack of social support is indicated by answering “yes” to “I lack enough support from family and friends”.

#### Appraisal

Appraisal includes the caregiver’s perception of caregiving, situational control, role conflicts, and the meaning of caregiving. In this study, appraisal was measured by three variables: whether the caregiver/care recipient has unmet needs and the score of role overload. The unmet needs perceived by caregivers are measured by whether they received and needed 14 support services for care recipients and 6 support services for caregivers. Role overload is measured by summing the score of whether caregivers report “yes” in managing the four areas: (1) Work, job; (2) Family, children; (3) Attend school; (4) Make enough money to live on, in addition to their role as an informal caregiver.

#### Exacerbating factors

Exacerbating factors refer to the caregiver’s physical health, including sleep problems, self-rated health, and performance of instrumental activities of daily living (IADL). Sleep problems are measured by the frequency of experiencing difficulty in falling asleep or staying asleep, waking up too early, restlessness, non-restful sleep in the last three days. The score ranges from 0 (not in the last three days) to 3 (daily in last three days). Self-rated health is measured by one question: “In general, how would you rate your health?”. The score ranges from 0 (excellent) to 3 (poor). IADL is measured by summing the scores of six activities related to independent living, with which the caregiver reported the level of assistance he/she needs (meal preparation, ordinary housework, managing finances, managing medications, shopping, and transportation).

### Outcomes: caregiving distress

InterRAI Chinese SCaN has five items to assess the caregiver’s self-perceived distress: (1) In the last 3 days, how often have you felt little interest or pleasure in things you normally enjoy? (2) In the last 3 days, how often have you felt anxious, restless, or uneasy? (3) In the last 3 days, how often have you felt sad, depressed, or hopeless? (4) In the last 3 days, how often have you felt overwhelmed by the Care Recipient’s condition? (5) In the last 3 days, how often have you felt unable to continue caring activities? Responses to each question were rated on a 4-point scale ranging from 0 to 3 points, and a summed total score (range 0–15 points) was calculated. A higher total score corresponds to a higher level of caregiver distress.

### Statistical analysis

Descriptive statistics were calculated to examine the demographics characteristics. Internal consistency of the interRAI Chinese SCaN scales was assessed using Cronbach’s alpha (α), with a value of 0.60 indicating acceptable internal consistency and more than 0.70 indicating good internal consistency [43]. Although Cronbach’s alpha is a widely used measure of reliability, McDonald’s omega coefficient (ω) relies on fewer and better realistic assumption/s and thus has been proved to be more robust than alpha [44, 45]. Thus, McDonald’s omega coefficient will also be reported. Construct validity, including concurrent validity and discriminant validity, were assessed using Pearson’s correlation (*r*) or Spearman rank correlations (*rho*) by correlating the interRAI Chinese SCaN scales and validation measures. To determine which scales would be appropriate for comparison, an in-depth evaluation of similar items, scales, and composites will be conducted during the content validation stage. A five-step hierarchical regression analysis will be applied to examine the predictive role of each domain in predicting caregiver distress. To evaluate multicollinearity among variables, the adjusted generalized standard error inflation factor (aGSIF) was used. All statistical analyses were performed using the R Studio. The level of statistical significance was set at *p* < .01 threshold (two-tailed).

## Results

### Sample characteristics

Table 1 presents descriptive statistics for the 531 caregivers’ profile. The final sample includes 50.47% Shanghai caregivers, 27.68% Hong Kong caregivers, 13.94% Singapore caregivers, and 7.91% Taiwan caregivers. Among all caregivers, 64.15% are female, with a mean age of 53.69 (SD = 15.8). The care recipients are 57.98% female, with a mean age of 77.08 (SD = 9.78). In this sample, 43.88% of caregivers are adult children, 17.7% are spouses or partners.

**Table 1:** Demographic characteristics (*N* = 531)

	M, SD	*N*, %
**Region**		
Shanghai		268 (50.47%)
Hong Kong		147 (27.68%)
Singapore		74 (13.94%)
Taiwan		42 (7.91%)
**CG age**	53.69 (15.80)	
**CG gender**		
Female		340 (64.15%)
Male		190 (35.85%)
**CR age**	77.08 (9.78)	
**CR gender**		
Female		305 (57.98%)
Male		221 (42.02%)
**Relationship with CR**		
Child or child-in-law		233 (43.88%)
Spouse/Partner/Significant other		94 (17.70%)
**Co-residence**		284 (53.48%)
**Marital status**		
Never married		136 (25.6%)
Married		360 (67.8%)
Partner/Significant other		10 (1.9%)
Widowed		7 (1.3%)
Separated		5 (0.9%)
Divorced		13 (2.4%)
**Years of providing care to the care recipient**		
1 month or less		28 (5.3%)
More than 1 month but less than 1 year		91 (17.1%)
1–5 years		173 (32.6%)
More than 5 years		239 (45%)
**Hours of care provided in the last three days to the care recipient**		
None		89 (16.8%)
Less than 3 h		69 (13%)
3 to less than 12 h		132 (24.9%)
12 to less than 24 h		60 (11.3%)
24 to less than 36 h		40 (7.9%)
36 h or more		141 (26.55%)
**Having financial difficulties**		193 (36.35%)
**Lack of social support**		164 (30.89%)
**CG having unmet needs**		359 (67.61%)
**CR having unmet needs**		298 (56.23%)
**Role overload (0–4)**	1.66 (1.59)	
**Having sleep problems (0–3)**	0.77 (0.95)	
**Self-rated health (0–3)**	1.23 (0.79)	
**CG IADL (0–12)**	1.14 (2.11)	
**Caregiver distress (0–15)**	3.27 (3.96)	
**Validation measurements**		
PHQ-4 (0–12)	2.82 (2.77)	
Pearlin role overload (4-16)	8.25 (3.06)	
LSNS-6 (0–30)	10.95 (5.8)	
**Survey completion method**		
Self-reported		370 (69.7%)
Assist by social workers		161 (30.3%)

Note. CG = Caregiver; CR = Care recipient; IADL = Instrumental activities of daily living; PHQ-4 = Patient Health Questionnaire-4; LSNS-6 = Lubben Social Network Scale-6

In the primary stressors, 53.48% of caregivers live together with the care recipient, and 26.55% provided more than 36 h of care in the past three days. In the secondary stressors, 36.35% of caregivers reported financial difficulties and 30.89% lack of support from family and friends. Regarding appraisal of the caregiving role, 67.61% of caregivers reported unmet supportive needs, primarily including episodic relief from caregiving and carer support groups. 56.23% of care recipients also have unmet supportive needs, mostly mental health services and physical rehabilitation. The mean score of role overload is 1.66 (SD = 1.59), indicating caregivers usually have difficulties managing one to two responsibilities in addition to the caregiving role. Finally, in terms of the exacerbating factor (physical health), the mean sleep problem score is 0.77 (SD = 0.95), the mean self-rated health score is 1.23 (SD = 0.79), and the mean score for IADL is 1.14 (SD = 2.11). Higher scores indicate worse physical health.

### Validation of the interRAI Chinese SCaN

#### Internal consistency reliability

The internal consistency of the two scales, which are summarized by more than 1 question, is evaluated by Cronbach’s alpha and McDonald’s omega coefficient. As shown in Table 2, the 2 composite measures in the translated assessment demonstrated strong inter-item reliability. The Cronbach’s alpha for caregiving distress and role overload is 0.91 and 0.83, respectively. The McDonald’s omega is 0.93 and 0.87, respectively, demonstrating more robust results.

**Table 2:** Internal consistency reliability, concurrent validity, discriminant validity of interRAI Chinese SCaN scales

interRAI Chinese SCaN scales	Inter-item consistency (α)	Inter-item consistency (ω)	Spearman’s correlation (concurrent validity)	Spearman’s correlation (discriminant validity)
Caregiving distress (5 items)	0.91	0.93	0.74*** (PHQ-4)	-0.2*** (LSNS-6)
Role overload (4 items)	0.83	0.87	0.45*** (Pearlin role overload)	-0.23*** (LSNS-6)

Note. α = Cronbach’s alpha; ω = McDonald’s omega; PHQ-4 = Patient Health Questionnaire-4; LSNS-6 = Lubben Social Network Scale-6

*** *p* < .0001

#### Face validity and content validity

Face validity was examined by all principal investigators in the four regions by assessing whether the items were (1) relevant to the Chinese caregiver’s needs (2), relevant to the context of informal caregiving in the Chinese population, and (3) suitable for informal Chinese caregivers in terms of acceptability and readability. The research team agreed that the items showed adequate face validity. Content validity was examined by comparing the translated and original items of the interRAI SCaN assessment, and the results suggest that the content validity is at an acceptable level. The scales that were compared were determined during the content validation stage based on the similarity between the definition and approach in developing the scales. Table 2 outlines the scales that were determined to be appropriate for comparison.

#### Concurrent validity and discriminant validity

The validity coefficients provided evidence of the scales’ construct validity. Given the non-normality of the distributions, correlations were determined using Spearman’s *rho*. The results in Table 2 indicate that the concurrent validity of interRAI Chinese SCaN scales were moderate to good (*rho* ranged from 0.45 to 0.74). The test between the two scales and LSNS-6 was also proved to be unrelated (*rho* ranged from − 0.23 to -0.2).

### Predictors of caregiving distress

Table 3 summarizes the results of the five-step hierarchical linear regression analyses that predicted caregiving distress. The adjusted generalized standard error inflation factor ranged from 1.04 to 1.44, providing evidence that there was no problem related to multicollinearity **(see Table S2)**.

**Table 3:** Hierarchical linear regression model for the effects of predictors on caregiving distress

Predictors	Model 1	Model 2	Model 3	Model 4	Model 5
**Background factors**					
Age	0.01*	-0.001	-0.003	-0.003	-0.002
Female	-0.05	-0.04	0.05	0.08	0.05
Spouse/partner as caregiver	0.24	0.19	0.2	0.15	0.001
Child as caregiver	0.12	0.05	-0.02	-0.05	-0.07
Region – Hong Kong (ref.)					
Region – Taiwan	-0.14	-0.1	0.19	0.09	0.03
Region – Singapore	-0.29*	-0.28*	-0.17	-0.17	-0.09
Region – Shanghai	-0.38***	-0.33***	-0.22*	-0.05	0.14
**Primary stressors**					
Co-residence		0.1	0.11	0.13	0.09
Informal care time > 36 h		1.27***	0.49***	0.24*	0.3**
**Secondary stressors**					
Financial difficulties			0.55***	0.15	0.05
Lack of social support			0.97***	0.64***	0.55***
**Appraisal**					
CR having unmet needs				0.39***	0.23*
CG having unmet needs				0.39***	0.3**
Role overload (0–4)				0.17***	0.14***
**Exacerbating factors**					
Sleep problems (0–3)					0.24***
Self-rated health (0–3)					0.09*
CG IADL (0–12)					0.12***
**Model statistics**					
F-statistic	2.832	11.44	24.49	28.93	33.52
*df*	7	9	11	14	17
*p*	0.007**	< 0.001***	< 0.001***	< 0.001***	< 0.001***
R-squared	0.04	0.1696	0.3492	0.449	0.5362
R-squared change	0.04	0.1296	0.1796	0.0998	0.0872

Note. CG = Caregiver; CR = Care recipient; IADL = Instrumental activities of daily living

*** *p* < .001; ** *p* < .01; * *p* < .05

The full model revealed a significant overall model fit, explaining 53.62% of the variance in caregiver distress. Entering the background factors, primary stressors, secondary stressors, appraisal, and exacerbating factors all significantly enhanced the model’s predictability, indicating that the source of caregiver distress is multidimensional. In the full model, caregivers with longer informal care time over 36 h in the past 3 days (*p* < .001), lack of support from family and friends (*p* < .001), have unmet needs (*p* < .01), experience role overload (*p* < .001), sleep problems (*p* < .001), and low IADL functioning (*p* < .001) are at a higher risk of caregiver distress.

## Discussion

Providing care at home is a highly demanding task both emotionally and physically, but informal caregivers are often a neglected and at-risk population [27]. This study was the first to translate and validate the Chinese version of the interRAI Self-reported Carer Needs (SCaN) assessment among the Chinese populations. Although the sample sizes in each region are slightly different due to the restraints of resources, we further calculated reliability and validity with sub-samples from each region, and the result shows good consistency (see Table S3). This study also sought to better understand caregiving distress across different patient conditions and caregiver contexts using the MCSB and hierarchical linear regression models. A sensitivity analysis was conducted with PHQ-4 as a measure of caregiving distress. The result shows no significant difference between these two models. It hypothesized that each domain of stressors (background factors of stress, primary stressors, secondary stressors, appraisal, and exacerbating factors) would significantly improve the predictability of the linear regression model for caregiving distress. The result confirmed this hypothesis as entering the five domains all significantly improved the model fit and demonstrated the multidimensionality of distress sources.

In the background factors, our result did not find informal caregiver’s age, gender, and relationship to the care recipients as predictors of caregiving distress. Nonetheless, from Model 1 to Model 3, Shanghai caregivers are significantly less distressed, which may contribute to the age differences in the four regions. In our sample, the mean age of the caregivers for Shanghai, Taiwan, Singapore, and Hong Kong caregivers are 61.36, 52.97, 50.57, and 41.55, respectively. Thus, Shanghai caregivers are mostly retired with fewer role conflicts, while Hong Kong caregivers are mostly in the working age. However, such differences diminished after adding factors on caregiving appraisal and physical functions as shown in Model 4 and Model 5.

In the primary stressors, co-residence is not a significant predictor of caregiver distress, which is consistent with previous research showing that Chinese caregivers are more resilient to the stress brought by cohabitation compared to Western caregivers as Chinese family members traditionally live together, and it is perceived as a way to fulfil their care responsibility [46]. We also found that informal care time significantly predicts caregiving distress across four models, especially those who provided more than 36 h in the past 3 days. This group comprised 26.55% of the caregivers in our sample, indicating the necessity to identify and provide respite and supportive services to the highly stressed caregivers with high caregiving intensity.

In the second stressor, the financial difficulty is only significant in Model 3, while the lack of support from family and friends remains significant and robust across Model 3 to Model 5. The findings largely mirrored previous studies demonstrating that sufficient family/social support is grounds for caregiver empowerment (e.g., 8).

In terms of appraisal of the caregiving role, caregivers’ unmet needs significantly escalate distress. Informal caregivers often need assistance and support to meet their physical, emotional, social, financial, and mental health needs to enable them to continue the caregiving role, but our result shows that 67.61% of Chinese caregivers have unmet needs. Meanwhile, caregivers with multifaceted roles such as employment, family, and education, in addition to their caregiving role, are at higher risk of experiencing distress.

Lastly, caregiver’s physical health is a robust predictor of caregiving distress, including their sleeping quality (*p* <. 001), IADL performance (p <. 001), and self-rated health (*p* <. 05), indicating caregivers are struggling to maintain their own health while providing care. While previous studies mostly focus on how the care recipient’s condition influences caregiving distress, few studies have explored the caregiver’s own health conditions. Older adult caregivers (age > 50 years older) particularly suffer from additional health risks due to insufficient time for self-care, the ageing process, and the high prevalence of chronic illnesses [47]. In our sample, the mean age of caregivers is 53.69 (SD = 15.8), and it is important to recognize the physical health needs and caregiving challenges they face to maintain their health and well-being. Otherwise, informal caregivers themselves will increasingly become recipients of care.

### Implications and limitations

The validation of the standardized assessment tool provides valid and reliable information and common grounds for international researchers to compare data cross-nationally. Early screening and routine assessment of caregiver distress should be part of the comprehensive care planning to address the collective needs of the care recipient–caregiver dyads. As an online anonymous survey, 70% caregivers completed it independently, demonstrating its feasibility and efficiency of caregiver self-report in clinical settings. In addition, influenced by the Confucian culture, Chinese caregivers may feel guilty for taking a break from the caregiving tasks and addressing their own needs. Therefore, healthcare practitioners should share the assessment result not only with the caregiver but also with their family, friends, and care recipients to help them validate their role and make their needs visible. In future, more targeted interventions and culturally appropriate support should be developed in alignment with the assessment outcomes.

The present study had four limitations. First, the study was cross-sectional; thus, the cause-and-effect relationships were not established, and it was not possible to explore the trajectory of caregiver distress over time. Longitudinal studies are required to overcome this limitation in the future. Second, caregivers were selected by convenience sampling method, which could result in selection bias and limit the generalizability of this study to other populations. The differences in sample sizes across sites can increase the risk of selection bias. Future work should investigate this further in a larger, representative sample to address the potential bias. Third, we did not conduct a test–retest exercise to determine the stability of caregiver responses. Fourth, as the interRAI SCaN assessment captures the caregiver’s characteristics, the care recipients’ conditions remain largely unknown (e.g., disease types, care dependency level, depressive levels, cognitive abilities). Therefore, we cannot address the care recipients’ factors contributing to the caregivers’ distress, such as Alzheimer’s disease and related dementias (ADRD) and behavioral and psychological symptoms of dementia (BPSD) have been proven major causes of caregiving distress for dementia care recipients (e.g., [48, 49]). Nor can we differentiate the caregivers’ distress by the acuity and severity of the disease. Future research can link the interRAI SCaN questionnaire and other interRAI instruments, such as interRAI Home Care, to examine caregiving distress more comprehensively. Caution is needed in the interpretation of our findings.

## Conclusion

In conclusion, our findings suggest that the interRAI Chinese SCaN assessment is a valid and reliable tool among Chinese informal caregivers of older adults. Healthcare professionals should early screen caregivers with longer informal care time, lack of support from family and friends, have unmet needs, experience role overload, sleep problems, and low IADL functioning, and provide supportive services across the caregiving journey.

### Electronic supplementary material

Below is the link to the electronic supplementary material.

Supplementary Material 1

## Data Availability

The datasets generated and/or analysed during the current study are available in the HKU Data Repository. The data are available from the authors upon reasonable request.

## Declarations

### Authors’ information

Prof. Vivian W. Q. Lou is the Director of the Sau Po Center on Aging, and a Professor at the Department of Social Work & Social Administration at the University of Hong Kong. Her research interests include family gerontology and evidence-based intervention.

## Abbreviations

SCaN: Self-reported Carer Needs
MCSB: Model of Carer Stress and Burden
PHQ-4: Patient Health Questionnaire-4
LSNS-6: Lubben Social Network Scale
PHQ-2: 2-item depression scale
GAD-2: 2-item anxiety scale
IADL: Instrumental activities of daily living
ADRD: Alzheimer’s disease and related dementias
BPSD: Behavioural and Psychological Symptoms in Dementia

## References

[1] Lindt N, van Berkel J, Mulder BC (2020). Determinants of overburdening among informal carers: a systematic review. BMC Geriatr.

[2] Lutz BJ, Tabloski PA, Turner TA (2022). Categorizing national caregiver recommendations to support family caregivers and address unmet needs. Nurs Outlook.

[3] Campione JR, Zebrak KA (2020). Predictors of unmet need among informal caregivers. Journals Gerontology: Ser B.

[4] Mollica MA, Smith AW, Kent EE (2020). Caregiving tasks and unmet supportive care needs of family caregivers: a US population-based study. Patient Educ Couns.

[5] Jika BM, Khan HTA, Lawal M (2021). Exploring experiences of family caregivers for older adults with chronic illness: a scoping review. Geriatr Nurs.

[6] Bell JF, Whitney RL, Young HM (2019). Family Caregiving in Serious Illness in the United States: recommendations to support an invisible workforce. J Am Geriatr Soc.

[7] Sun Y, Iwagami M, Watanabe T, Sakata N, Sugiyama T, Miyawaki A, Tamiya N (2021). Factors associated with psychological distress in family caregivers: findings from nationwide data in Japan. Geriatr Gerontol Int.

[8] George ES, Kecmanovic M, Meade T, Kolt GS (2020). Psychological distress among carers and the moderating effects of social support. BMC Psychiatry.

[9] Bom J, Bakx P, Schut E, van Doorslaer E (2019). The impact of Informal Caregiving for older adults on the health of various types of caregivers: a systematic review. Gerontologist.

[10] Maxwell CJ, Campitelli MA, Diong C, Mondor L, Hogan DB, Amuah JE (2018). Variation in the health outcomes associated with frailty among home care clients: relevance of caregiver distress and client sex. BMC Geriatr.

[11] Tsai W-C, Lin H-C, Chang C-C, Chang W-N, Huang C-C, Cheng K-Y (2020). Neuropsychiatric symptoms in Parkinson’s disease: association with caregiver distress and disease severity. Int Psychogeriatr.

[12] Burhanullah MH, Munro CA (2020). Influences on caregiver distress in Asia: a moving target. Int Psychogeriatr.

[13] Kirk DL, Kabdebo I, Whitehead L (2022). Prevalence of distress and its associated factors among caregivers of people diagnosed with cancer: a cross-sectional study. J Clin Nurs.

[14] Pauley T, Chang BW, Wojtak A, Seddon G, Hirdes J (2018). Predictors of Caregiver Distress in the community setting using the Home Care Version of the Resident Assessment Instrument. Prof case Manage.

[15] He W, Goodkind D, Kowal P, Almasarweh IS, Giang TL, Islam MM et al. Asia Aging: Demographic, Economic, and Health Transitions. 2022.

[16] Asian Development Bank. Asian Development Outlook (ADO) 2023 [ https://www.adb.org/outlook/editions/september-2023.

[17] Isac C, Lee P, Arulappan J (2021). Older adults with chronic illness – caregiver burden in the Asian context: a systematic review. Patient Educ Couns.

[18] Liu Z, Sun W, Chen H, Zhuang J, Wu B, Xu H (2022). Caregiver burden and its associated factors among family caregivers of persons with dementia in Shanghai, China: a cross-sectional study. BMJ open.

[19] Yung LYY, Fan R (2015). The east Asian family-oriented Principle and the Concept of Autonomy. Family-oriented informed consent: east Asian and American perspectives. Philosophy and Medicine.

[20] Nie J-B (2015). The Benevolent polity: a confucian socio-ethical vision of Eldercare. Asian Bioeth Rev.

[21] Yan E, Chan K-L, Tiwari A (2015). A systematic review of prevalence and risk factors for elder abuse in Asia. Trauma Violence Abuse.

[22] Ng HY, Griva K, Lim HA, Tan JYS, Mahendran R (2016). The burden of filial piety: a qualitative study on caregiving motivations amongst family caregivers of patients with cancer in Singapore. Psychol Health.

[23] Brandão D, Ribeiro O, Oliveira M, Paúl C (2017). Caring for a centenarian parent: an exploratory study on role strains and psychological distress. Scand J Caring Sci.

[24] Niu A, Guo C, Zhong D, He G, Zhong W, Wang L (2021). Identifying the Unmet supportive care needs, with concomitant influencing factors, in Family caregivers of Cancer patients in China. Asia-Pacific J Oncol Nurs.

[25] Davidson PM, Abshire MA, Paull G, Szanton SL (2018). Family caregivers: important but often poorly understood. J Clin Nurs.

[26] Abey-Nesbit R, Doren S, Ahn S, Iheme L, Peel NM, Declercq A (2022). Factors associated with caregiver distress among home care clients in New Zealand: evidence based on data from interRAI Home Care assessment. Australas J Ageing.

[27] Sherman DW (2019). A review of the Complex Role of Family Caregivers as Health Team members and second-order patients. Healthcare.

[28] Hallikainen I, Koivisto AM, Välimäki T (2018). The influence of the individual neuropsychiatric symptoms of people with Alzheimer disease on family caregiver distress—A longitudinal ALSOVA study. Int J Geriatr Psychiatry.

[29] Teixeira MJC, Abreu W, Costa N, Maddocks M (2020). Understanding family caregivers’ needs to support relatives with advanced progressive disease at home: an ethnographic study in rural Portugal. BMC Palliat care.

[30] Pearlin LI. The sociological study of stress. J Health Soc Behav. 1989:241–56. 2674272

[31] Pearlin LI, Mullan JT, Semple SJ, Skaff MM (1990). Caregiving and the stress process: an overview of concepts and their measures. Gerontologist.

[32] Singapore Department of Statistics. Singapore Department of Statistics. Census of Population 2020 Statistical Release 1 - Key Findings. In: Statistics SDo, editor. 2020.

[33] Lin J-P, Yi C-C (2019). Dilemmas of an Aging Society: family and state responsibilities for intergenerational care in Taiwan. J Fam Issues.

[34] Lien YF, Huang HM (2017). Challenges in intergenerational caregiving for frail older people: a multiple case study. Nurs Health Sci.

[35] Liu Y, Hughes MC, Roberto KA, Savla J (2022). Physical and mental health of family caregivers of older parents and grandchildren in China. Aging Health Res.

[36] Yiu HC, Zang Y, Chew JHS, Chau JPC (2021). The influence of confucianism on the perceptions and process of caring among family caregivers of persons with dementia: a qualitative study. J Transcult Nurs.

[37] Brislin RW, Freimanis C. Back-translation: a tool for cross-cultural research. In: Chan S-W, Pollard DE, editors. An Encyclopaedia of translation: Chinese-English, english-chinese translation: Hong Kong. Chinese University.; 2001. pp. 22–40.

[38] Kroenke K, Spitzer RL, Williams JBW, Lowe B (2009). An Ultra-brief Screening Scale for anxiety and depression: the PHQ-4. Psychosomatics.

[39] Fong TCT, Ho RTH, Yip PSF (2023). Psychometric properties of the Patient Health Questionnaire-4 among Hong Kong young adults in 2021: associations with meaning in life and suicidal ideation. Front Psychiatry.

[40] Pearlin Role Overload Measure (Pearlin ROM) [Database record]. APA PsycTests. 1990. https://psycnet.apa.org/doiLanding?doi=10.1037%2Ft36880-000.

[41] Cheng S-T, Lam LCW, Kwok T, Ng NSS, Fung AWT (2013). Self-efficacy is Associated with Less Burden and more gains from behavioral problems of Alzheimer’s Disease in Hong Kong Chinese caregivers. Gerontologist.

[42] Chang Q, Sha F, Chan CH, Yip PSF (2018). Validation of an abbreviated version of the Lubben Social Network Scale (LSNS-6) and its associations with suicidality among older adults in China. PLoS ONE.

[43] Nunnally JC, Bernstein IH. The Assessment of Reliability. Psychometric Theory. 1994;3:248–92.

[44] McDonald RP (1999). Test theory: a unified treatment. Mahwah.

[45] Bonniga R, Saraswathi DA. Literature Review Of Cronbachalphacoefficient And Mcdonald’s Omega Coefficient. Eur J Mol Clin Med. 2020;7(06).

[46] Chan CY, Cheung G, Martinez-Ruiz A, Chau PYK, Wang K, Yeoh EK, Wong ELY (2021). Caregiving burnout of community-dwelling people with dementia in Hong Kong and New Zealand: a cross-sectional study. BMC Geriatr.

[47] Sabo K, Chin E (2021). Self-care needs and practices for the older adult caregiver: an integrative review. Geriatric Nurs (New York).

[48] Mukherjee A, Biswas A, Roy A, Biswas S, Gangopadhyay G, Das Shyamal K (2017). Behavioural and psychological symptoms of dementia: correlates and impact on Caregiver Distress. Dement Geriatric Cogn Disorders Extra.

[49] Wang J, Xiao LD, Li X, De Bellis A, Ullah S (2015). Caregiver distress and associated factors in dementia care in the community setting in China. Geriatr Nurs.

