# Supplementary material for: Validating interRAI Chinese self-reported carer needs (SCaN) assessment and predicting caregiving distress among informal Chinese caregivers of older adults
Source: BMC Geriatr. 2024 May 8;24:409. doi: 10.1186/s12877-024-05014-0 (PMC11080141; doi:10.1186/s12877-024-05014-0)
Supplement: Supplementary file 1 — Supplementary Material 1 [file 12877_2024_5014_MOESM1_ESM.docx]

**Supplementary Tables**

**Table S1**

Description and Supporting Evidence of the 12 Factors Used in the Model

| **Background factors of stress** | Age | Young caregivers have been found to have higher rates of distress due to their competing demands of work, childrearing, and eldercare (Sun *et al.*, 2021). However, competing evidence shows that older age is associated with higher caregiver distress due to age-related health risks (Pauley *et al.*, 2018). |
| --- | --- | --- |
|  | Gender | Female caregivers provide more informal care and are more likely to be distressed than male caregivers (Kirk *et al.*, 2022; Sun *et al.*, 2021). |
|  | Relationship to care recipient | Higher odds of caregiver distress have been observed when a spouse is a primary caregiver (Abey-Nesbit *et al.*, 2022; Penning and Wu, 2016). Adult children who were primary caregivers were also likely to experience caregiver distress, as they have multifaceted responsibilities (Chan *et al.*, 2021; Isac *et al.*, 2021). |
| **Primary stressors** | Co-residence | Living with care recipients has been found to increase the odds of caregiver distress in Western societies, but not among Chinese family caregivers (Chan *et al.*, 2021; Pauley *et al.*, 2018). |
|  | Informal care time | Longer caregiving time and intensity are associated with risk of caregiver distress (Abey-Nesbit *et al.*, 2022; Sun *et al.*, 2021). |
| **Secondary stressors** | Financial difficulties | Perceived income inadequacy can predict psychological distress in Alzheimer’s caregivers (Sun *et al.*, 2009). |
|  | Social support | Social support directly affects the psychological health among family caregivers of frail older people (del-Pino-Casado *et al.*, 2022). Less emotional support was associated with higher levels of distress (Miller *et al.*, 2001). |
| **Appraisal** | CG and CR unmet needs | Temple (2018) found that caregivers having any unmet needs increased the odds of psychological distress by twofold. Maxwell *et al.* (2018) pointed out that despite the ongoing calls for public services, the significant unmet needs of caregivers and care recipients remain a lingering concern. |
|  | Role overload | Role strains, specifically role captivity, constitute an important facet of caregiving distress (Brandão *et al.*, 2017). |
| **Exacerbating factors** | Sleep problems | Morris (2015) reported that insomnia symptoms are common in cancer caregivers who call helplines and are related to distress. |
|  | Self-rated health | Caregiver’s own poor health status was associated with psychological distress (Brandão *et al.*, 2017; Sun *et al.*, 2021). |
|  | Caregiver’s IADL | Health problems were associated with psychological distress. Van Hof *et al.* (2022) reported that 30% of the caregivers had reduced physical functioning in a sample of cancer caregivers. |

**Table S2**

Multicollinearity

|  | **GVIF** | **DF** | **GVIF^(1/(2*Df))** |
| --- | --- | --- | --- |
| **CG age** | 1.98 | 1 | 1.41 |
| **CG gender** | 1.09 | 1 | 1.04 |
| **Spouse/partner as caregiver** | 2.08 | 1 | 1.44 |
| **Child as caregiver** | 1.43 | 1 | 1.19 |
| **Region** | 2.49 | 3 | 1.16 |
| **Co-residence** | 1.4 | 1 | 1.18 |
| **Informal care time** | 1.51 | 1 | 1.23 |
| **Financial difficulties** | 1.79 | 1 | 1.34 |
| **Lack of social support** | 1.62 | 1 | 1.27 |
| **CR having unmet needs** | 1.92 | 1 | 1.39 |
| **CG having unmet needs** | 1.89 | 1 | 1.38 |
| **Role overload** | 1.98 | 1 | 1.41 |
| **Sleep problem** | 1.5 | 1 | 1.22 |
| **Self-rated health** | 1.47 | 1 | 1.21 |
| **CG IADL** | 1.22 | 1 | 1.1 |

*Note.* CG=Caregiver; CR=Care recipient

**Table S3**

Reliability and validity with sample in each region

|  | **Caregiving distress (5 items)** | | |
| --- | --- | --- | --- |
|  | Inter-item consistency (α) | Inter-item consistency (ω) | Spearman’s correlation |
| **Shanghai (n=268)** | 0.94 | 0.97 | 0.75*** |
| **Hong Kong (n=147)** | 0.86 | 0.9 | 0.72*** |
| **Singapore (n=74)** | 0.9 | 0.94 | 0.75*** |
| **Taiwan (n=42)** | 0.8 | 0.88 | 0.73*** |

*** *p*<.001

|  | **Role overload (4 items)** | | |
| --- | --- | --- | --- |
|  | Inter-item consistency (α) | Inter-item consistency (ω) | Spearman’s correlation |
| **Shanghai (n=268)** | 0.91 | 0.93 | 0.48*** |
| **Hong Kong (n=147)** | 0.68 | 0.76 | 0.4*** |
| **Singapore (n=74)** | 0.58 | 0.69 | 0.45*** |
| **Taiwan (n=42)** | 0.75 | 0.88 | 0.1 |

*** *p*<.001
